# Bioprinting techniques for regeneration of oral and craniofacial tissues: Current advances and future prospects

**DOI:** 10.1016/j.jobcr.2025.01.019

**Published:** 2025-02-12

**Authors:** Shailesh Varshney, Anshuman Dwivedi, Vibha Pandey

**Affiliations:** a Department of Periodontology, School of Dental Sciences, Sharda University, Greater Noida, Uttar Pradesh, India; b,Department of Stem Cells & Regenerative Medicine, Santosh, University, Ghaziabad, Uttar Pradesh, India; c,Department of Psychology, Himalayan, Garhwal University, Uttarakhand, India

**Keywords:** Bioprinting, Bioink, Bioscaffold, Microspheroid, Bioprinter, Hydrogel

## Abstract

**Background:**

Regenerative dentistry aims to reinstate, fix, renew, and regrow tissues within the oral and craniofacial domain. Existing regenerative methods are based on insights into tissue biology or disease processes that lead to tissue degradation. However, achieving complete and functional Tissue regeneration remains a primary challenge in real-world medical scenarios.

**Aim:**

The review focuses on the application of bioprinting techniques for rejuvenating intricate Oral and craniofacial tissues, such as craniofacial bone, periodontal ligament, cementum, dental pulp, temporomandibular joint cartilage, and whole teeth.

**Methods:**

Bioprinting, a cutting-edge technology in regenerative dentistry, strives to create entirely new Functional tissues and organs. This approach merges principles from engineering and biology to produce three-dimensional biologically operational constructs containing bioactive substances, Living cells and cell clusters using automated bioprinters. The review summarizes the outcomes achieved through bioprinting techniques in both in vitro (laboratory experiments) and in vivo (Studies on living organisms) experiments.

**Result:**

The emergence of this innovative tissue engineering technology has yielded highly promising outcomes during the experimental stages.

**Conclusion:**

These promising experimental results necessitate replication through human clinical trials to ascertain the viability of bioprinting techniques for mainstream clinical implementation in regenerative dentistry.

## 1 Introduction

A tooth, characterized as an intricate biological organ, is composed of several distinct tissues enamel, dentin, cementum, and pulp. It is embedded within a sophisticated structure known as the periodontium, which encompasses cementum, periodontal ligament (PDL), bone, and gingiva.^1^ The combination of a tooth and its supporting periodontal tissues is commonly referred to as the tooth alveolar bone unit with an epithelial and mesenchymal origin. This tooth-alveolar bone unit is one of the crucial component of craniofacial complex. This complex also encompasses other vital structures like cartilage, muscles, ligaments, blood vessels and nerves.^2^ Various disease processes, spanning from dental caries to trauma to complex periodontal diseases, can damage or compromise these tissues and associated structures in the craniofacial region.^3^^,^^4^ The primary objective for most clinicians is to reinstate these structures to their functional state. Depending on the circumstances, clinicians employ both conventional and advanced therapies. Advanced treatments designed to regenerate tissues and structures encompass stem cell therapy, the utilization of scaffolds loaded with stem cells, morphogens/growth factors, nanotechnology, and gene therapy.^5^^,^^6^ Alongside these cutting edge therapies, clinicians also use the potential of 3-D printing technology.^7^ This technology is rapidly gaining traction in the healthcare field, including medicine and dentistry. Within dentistry, it serves a multitude of purposes, such as aiding in dental implants for guide drill preparation, fabrication of stereolithography (STL) models via rapid prototyping technique, generating physical models, constructing dental, craniomaxillofacial, and orthopedic implants, and fabricating copings and frameworks for implant and dental restorations.^8^ Analogous to 3-D printing, there exists the technique of biofabrication or bioprinting. This method aims to produce 3-D functional tissue constructs to regenerate intricate biological structures, utilizing raw materials such as living cells, matrices, biomaterials, and molecules.In this process, 3-D functional tissue constructs are autonomously created using a substance termed bioink. Bioink is composed of either a single bio-material or a combination of several biomaterials, in the form of hydrogels. These hydrogels are subsequently seeded with specific cell types according to the tissue intended for regeneration by the clinician.^9^ Seed cells need to possess optimal capabilities for both proliferation and the secretion of extracellular matrix (ECM). A variety of seeding methods, including spinner flask, orbital shaker, static seeding, negative pressure seeding, centrifugal seeding, and perfused bioreactor, can be employed to improve the seeding of cells onto scaffolds and facilitate efficient tissue regeneration.^10^ The biomaterial within bioinks comprises natural or synthetic materials, or at times, solely cell aggregates without any biomaterial (microspheroids, μS).^11^ Bioinks derived from natural sources encompass gelatin, alginate, fibrin/fibrinogen, collagen, hyaluronic acid, hydroxylapatite, chitosan, gellan gum, silk, agarose, decellularized extracellular matrix (dECM), while synthetic materials include substances like poly(ethylene glycol) (PEG), polycaprolactone (PCL), and Pluronic. The fabrication of the desired 3-D constructs is accomplished using a device termed a Bioprinter. Bioprinters are categorized into three classes based on the mechanism employed for biofabrication: Droplet-based bioprinters (DBB) Laser-based bioprinters (LBB) and Extrusionbased bioprinters (EBB).^12^ In the process of tissue engineering through bioprinting, the initial step involves the careful selection of specific cells, designated as seed cells. These cells are then combined with bioactive additives on a biodegradable scaffolds to create artificial grafts, which are then implanted into the intended defect site.^9^ Cell proliferation and matrix secretion happen concurrently with the breakdown of the scaffold material, resulting in the formation of new tissues and regional anatomical structures. At present, within the medical field, the 3-D bio-fabrication technique is predominantly employed for simulating and reconstructing hard tissues, such as long bones and the spine, as well as for designing drug-delivery systems.^7^ It is also applied to regenerate various tissues, including the cardiac, neural, cartilage, and vascular tissues. Although still in the early phases of research and development, this engineering tool holds immense promise in the realm of tissue and organ regeneration, especially for intricate tissue organs. The cranio-facial region offers a wealth of tissues characterized by complex biphasic and multiphasic structures, making the bioprinting technique a potential solution for restoring function to diseased and damaged tissues. Thus, the authors of this paper endeavor to elucidate the application of the 3-D bioprinting process of constructing scaffolds for the regeneration or reconstruction of complex biphasic and multiphasic tissues in the oral and craniofacial regions. This paper reviews the challenges and limitations associated with traditional regenerative processes for specific oral and craniofacial tissues, the creation of bioprintable scaffolds utilizing appropriate bioink and bioprinter,the rationale behind incorporating cells or bioactive cues into the constructed scaffold, and the utilization of this scaffold in in vitro or in vivo studies, accompanied by the resultant outcomes in regenerating distinct oral and craniofacial tissues.

## 2 Three-dimensional bioprinting for the regeneration of craniofacial bone

The periosteum, compact bone, cancellous bone, and the cribriform plate that houses tooth sockets are the four main layers of the alveolar bone, a pliable ridge in the jaw that supports dental structures and tooth sockets.^13^ This structure exhibits reactivity to a variety of stimuli, most notably tooth loss resulting in resorption, and poses serious obstacles to treatment strategies such as implant insertion. The alveolar bone, like other bones, is highly mineralized, primarily with semicrystalline hydroxyapatite. Its structure comprises a 70 % organic matrix, primarily collagen type I. The cellular composition ranges from 2 to 5 %, mainly comprising bone-lining cells, osteoblasts, osteoclasts, and osteocytes.^14^ Various methods have been employed for bone regeneration, such as the use of autografts,^15^ allografts^15^ and ceramic/polymeric scaffolds^16^ for the management of bony defects. Nevertheless, these methods encounter several difficulties in clinical settings, such as: i) immunological reactions and infections associated with allografts,^17^ ii) morbidity at the donor site and scarcity of autograft tissue, especially bone,^15^ and iii) insufficient control at the hard/soft tissue interface in scaffolds manufactured using conventional methods, which limits their effectiveness.^18^ The most complex clinical challenge involves repairing composite CMF (Craniomaxillofacial) defects, which requires precise, layered placement of multiple tissues to mimic the native tissue's anatomy and function. The intricate form of the skull demands meticulous attention, with diverse contour zones in the cranium, where convex and concave elements interplay, posing distinct challenges in reconstruction. To address these challenges, numerous researchers have endeavored to regenerate dentoalveolar and craniofacial bone by employing 3D scaffolds comprising bioinks containing both natural and synthetic polymers, and encapsulating stem cells. Creating a bioink for tissue engineering in the alveolar bone region involves considering its variations from other bones in the body, particularly regarding functional activities such as the applied load during facial physical activities.^19^ As a result, the bioink needs to take into account the bioactivity of different kinds of cells as well as variations in the mechanical qualities of various bone layers. Young's modulus, a critical mechanical parameter that must be replicated, has values of roughly 0.9 ± 109 N/m2 in cancellous alveolar bone and 13.7 ± 109 N/m2 in highdensity alveolar bone.^20^ Bioinks derived from natural polymers and similar components, are particularly appealing options for tissue engineering in the alveolar bone region because they have the ability to replicate the mechanical properties of alveolar bone while accommodating various cell types(Table 1). Collagen being not only an essential component of extracellular matrix (ECM) but also highly biocompatible is commonly used in tissue engineering applications. Collagen has the ability to create hydrogels that are suitable for cell encapsulation. Studies in the dentoalveolar field have demonstrated that the cell viability of dental pulp stem cells (DPSCs) encased in collagen type I and II exceeds 95 %. Nevertheless, collagen having comparatively inferior mechanical properties mean that in order to make it appropriate for bioprinting, it frequently needs to be crosslinked, mixed or modified with other materials.^21^^,^^22^ Other modifications of collagen-based bioinks include using a dental-specific bioink containing hDPSCs or incorporating gene-activated matrices for controlled co-delivery of growth factors**.**^23^This was accomplished by physically combining collagen type-I, or dECM, produced from pig bone, with an adequate concentration of β-TCP (β-Tri-calcium phosphate) to form a biocomposite that was loaded with cells.^21^ It was assumed that the osteoinductive characteristics of β-TCP and the tissue-specific biochemical signals from dECM would work in concert to influence the cell proliferation and osteo/odontogenic differentiation of hDPSCs in the printed dental structures. In-vitro evaluations that included osteo/odontogenic gene expression, calcified tissue matrix, and cell viability and growth were carried out in order to verify the printed biocomposite in comparison to a control that consisted of collagen/β-TCP bioink loaded with hDPSCs. Eight weeks after transplantation, the hDPSC-loaded biocomposite was inserted into the mice's subcutaneous tissue, exhibiting its capacity to cause ectopic hard tissue formation with osteo/odontogenic properties.^21^ A unique bioink called "hard tissue ink (HT-ink)" was introduced for intra-operative bioprinting (IOB) or in-situ/in-vivo bioprinting purposes, with the goal of reconstructing craniofacial abnormalities through the regeneration of composite tissues such as skin, muscle, and bone.^22^ High concentrations of collagen, chitosan (CS), nano-hydroxyapatite particles (nHAp), and β- Glycerophosphate disodium salt hydrate (β-GP) were present in the HT-ink composition. More specifically, mesenchymal stem cells were made to differentiate by using β-GP as an agent to induce osteogenesis.^24^ Furthermore, when the collagen was neutralized at 37 °C, effective collagen fibrillogenesis and physical crosslinking took place, producing a thicker and stronger fibrous structure.^25^, ^26^ The same author^23^ introduced another modification to intra-operative bioprinting (IOB) by incorporating gene-activated matrices into an osteogenic bioink. This strategy used a geneactivated matrix, an osteogenic bioink loaded with plasmid-DNAs (pDNA) to promote bone regeneration in conjunction with controlled co-delivery of growth factors. Plasmid-DNA encoding platelet-derived growth factor-B (pPDGF-B) and chitosan nanoparticle-encapsulated plasmid-DNA encoding bone morphogenetic protein-2 (CS-NPs(pBMP2)) were combined in this regulated co-delivery approach. In vitro, this combination produced a steady release of pBMP-2 for five weeks and a quick release of pPDGF-B within ten days. In order to transfect host cells and enable the encoded BMP-2 to stimulate the differentiation of recruited endogenous progenitors into the osteogenic lineage and improve bone regeneration in vivo, pBMP-2 was released with a prolonged and delayed duration.^23^ Hyaluronic acid (HA) is another naturally occurring polymer widely used in this sector. It is a glycosaminoglycan (GAG) that is widely distributed in the ECM of different soft tissues. It doesn't cause thrombosis or inflammation in the host tissues and is biocompatible and biodegradable. Owing to these qualities as well as its shear-thinning properties, it has been extensively used in tissue engineering and bioprinting techniques.^27^ But HA, like other hydrogels made from natural sources, demonstrates weak mechanical properties that make printing of stable structures difficult. As a result, in order to guarantee extended mechanical integrity, post-printing shape fidelity, and controlled degrading behavior, crosslinking or chemical treatment is often necessitated. These changes have allowed for the bioprinting of several tissues, such as bone, cartilage, and vascular tissue, using HA.^28^ In the realm of dentoalveolar regeneration, hyaluronic acid-based materials have also been employed as hydrogel-encapsulating cells. These materials aim to regenerate both alveolar and craniofacial bone, incorporating various strategies to enhance their mechanical properties. The proposed method for regenerating alveolar bone involved the combination of HA with a gelatin thiol derivative (gelatin-DTPH). This mixture was crosslinked using polyethylene glycol diacrylate, and polycaprolactone was employed as a supportive material. The initial venture into bioprinting with HA involved the use of a bioink along with an extrusionbased bioprinter. This was achieved by combining HA with gelatin, fibrinogen, and glycerol, with the goal of regenerating alveolar bone. In this process, a cell-laden hydrogel was extrusionprinted alongside a polycaprolactone support. Notably, the introduction of a sacrificial material, Pluronic F127, was first reported, enhancing the precision of the printing.^29^ In a unique approach distinct from the combination of HA with other substances, the study conducted by Kuss et al.,^30^ **i**ntroduced a significant innovation by modifying HA with methacryloyl groups. This modification showcased its potential to be utilized in conjunction with gelatin methacrylamide (GelMA) and polycaprolactone for the bioprinting of alveolar bone. The study highlighted the importance of minor molecular modifications and the use of HA derivatives, in the effectiveness of bioprinting techniques. A bioink utilizing HA was documented for the construction of a 3D bioprinted hydrogel laden with osteocytes, with the aim of achieving biomimetic mineralization in vitro. With a dense cell population of 10^7^ cells per milliliter and high cell survival of 85–90 %, the resultant construct demonstrated impressive shape fidelity. The bioinks were composed of biomimetic modified biopolymers, namely GelMA and hyaluronic acid methacrylate (HAMA), either with or without type I collagen added to it.^31^ To ensure appropriate printability and produce a dental-specific microenvironment that supports the development of hDPSCs, a newly designed bioink was developed that incorporated HA and a BMP-mimetic peptide for 3D bioprinted bioengineered dental tissue construction. The in-vitro results showed that, the BMP-GelMA bioink formulation significantly increased hDPSC survival and proliferation while hastening their odontogenic differentiation in the 3D bioprinted constructs.^32^ Furthermore, fibrin, a glycoprotein composed of fibrinogen monomers, known for their ability to enhance cell adhesion and proliferation, have also been utilized in bioprinting approaches for the regeneration of dental tissue and have shown promising results in the field of tissue engineering Fibrin is a good choice for dentoalveolar tissue engineering since it has been shown to support vascularization in dental tissues and to help differentiate DPSCs into odontoblasts.^33^ However, fibrin-based tissue engineering techniques do face the obstacles like gel shrinkage, poor mechanical stability, and rapid deterioration. An integrated tissue-organ printer (ITOP) was used to regenerate the mandible and calvarial bone using a bioink made of gelatin, fibrinogen, HA, and glycerol.^29^ The goal of adopting ITOP was to create a range of shapes, stable, human-scale tissue structures. Alginate is another naturally occurring polymer that is biocompatible and nonimmunogenic. It can easily dissolve in water and form hydrogels via ionic cross-linking. By maximizing polymer density and crosslinking density, its properties, including compressive modulus, stiffness, and gelation rate, can be easily be altered.^34^ Alginate, however, does have a major disadvantage that it lacks biological cues that could prevent cell attachment and may result in cell death. Numerous strategies, such as mixing alginate with other materials or altering it with cell-binding peptides, have been put forth to address this problem. Tian et al.,^35^ in their study employed a method that included utilizing of a composite hydrogel of sodium alginate, gel, and nano-hydroxyapatite (SA/Gel/na-HA) as a bioink and human periodontal ligament stem cells (hPDLSCs). Through this they 3D printed SA/Gel/na- HA/hPDLSCs cell bioscaffolds and evaluated the in-vitro biological features of this bioink, such as bioscaffold adherence, live/dead staining, cell proliferation, and osteogenic differentiation. According to the results, the SA/Gel/na-HA composite hydrogel had good rheological characteristics, making it ideal for printing. The inclusion of na-HA enhanced the composite bioscaffold's compressive qualities, and the scaffold showed a high swelling rate, suggesting sufficient porosity. Good biocompatibility was demonstrated by the bioscaffold, This encouraged hPDLSCs to differentiate into osteoblasts. Consequently, it had the potential to serve as a novel material for bone regeneration in tissue engineering applications. While natural polymers are ideal for tissue engineering due to their favorable biological response,^36^ they often encounter the drawback of poor mechanical properties. This limitation compromises their reliability in the printing process and their ability to regenerate hard tissues like bone. In contrast, synthetic polymers provide a high level of control over various physicomechanical properties, including the compressive modulus and degradation rate. This is attributed to the tunable nature of the synthesis procedure, which involves the assembly of monomers into distinct blocks. Nonetheless, a significant obstacle faced by the majority of synthetic polymers is addressing their deficiency in cell adhesion sites.This means that these molecules must be combined with natural polymers or those incorporated into polymer backbones. Various synthetic materials, including poly (hydroxyethyl methacrylate) (pHEMA), PEG, PCL, Poloxamer 407 (Pluronic F127), and poly (N-isopropylacrylamide) (pNIPAM), have been utilized for this purpose. Consequently, these synthetic polymers are commonly blended with natural polymers to formulate a bioink with enhanced biological and mechanical properties to create 3D bioprintable scaffolds. Authors, such as Kang et al. (2016),^29^ Kuss et al. (2017),^30^ Park et al.(2020),^32^ Chimene et al. (2020),^37^ and Yang et al. (2020)^31^ have developed such Composite bioinks for the regeneration of alveolar and craniofacial bones.

**Table 1:** Design Features of Studies Utilizing 3-D Bioprinting for regenerating Craniofacial bone.

*References*	*Bio-ink*			*Cell Density*	*Assessments*	*Tissue Regeneration*	*Young’s Modulus of Elasticity*	*Cell Viability*	*Shape*	*Dimension*	*Bio-printed Construct*
	Scaffold Material	Cell Seed	Bioactive Factor(BF)								
**Moncal et al., 2022****^23^**	Collagen + chitosan + β-glycerophosphate + nHAp	Primary rBMSCs	pPDGF-B and pBMP2	8 x10^5^ cells/mL	***In-vitro*** 1. Cell viability 2. Cell proliferation 3. Calcium deposition 4. Protein expression 5. Gene expression 6. Immunofluorescence Staining ***In-vivo*** Bone regeneration using μCT 1.BV/TV %, 2.BMD % 3.Bone coverage area (%) .	Bone regeneration in calvarial defects (Critical size defects)	----	>95%	Cylindrical	….	Bio-printed Bone Construct
**Kim et al., 2022****^21^**	Collagen type-I+ β-TCP	hDPSCs	….	1 x 10^7^ cells/ml	***In-vitro*** 1.Cell viability and cell proliferation. 2.Growth of calcified tissue matrix 3. ALP , OPN, and DSPP expressions 4.Osteo/odontogenic gene expression. ***In-vivo*** 1.Histology and immunohistochemistry examination for ectopic tissue formation.	Osteo/odontogenic ectopic hard tissue formation in Subcutaneous tissue of Mice	27.9 ± 2.2 kPa	95.8% ± 0.3%	----	8 x 8 x 4 mm^3^	
**Moncal et al., 2021****^22^**	Collagen + chitosan +β -glycerophosphate + nHAp	rBMSCs	rhBMP-2	5 x 10^6^ cells/mL	***In-vitro*** 1. Cell viability 2. Cell proliferation 3.Compression testing 4.Gene expression ***In-vivo*** .Bone regeneration using μCT 1BV/TV) % 2.Normalized BMD % 3. Bone coverage area (%)	Calvarial defects (Composite Tissue Regeneration)	8.2 ± 1.4 kPa	>95%	Cylindrical	Diameter-6mm and 5mm Height :Not Mentioned	
**Tian et al., 2021****^35^**	Alginate + gelatin + nHAp	hPDLSCs	----	----	***In-vitro*** 1. Cell viability 2. Cell adhesion 3. Cell proliferation 4. ALP activity 5. Compression properties	Osteogenic differentiation	8.27 MPa	----	Square	----	
**Chimene et al., 2020**^**37**^	GelMA + kCA + nSi (NICE bioink)	Bone marrow derived hMSCs	----	2500 cell/cm^2^	***In-vitro*** 1.Mechanical characteristics. 2.Cell-assisted matrix remodeling (histological) 3. Calcium content 4.Gene expression.	Osteogenic differentiation of encapsulated cells.	40±17 kPa to 103±6 kPa.	----	Hollow tube	Height-3mm Outer Diameter-10mm (c) Wall thickness-1mm	
**Park et al., 2020****^32^**	GelMA	hDPSCs	Synthetic BMP-2 mimetic peptide.	…..	***In-vitro*** 1. Cell viability 2. Cell proliferation 3. Osteogenic differentiation 4. Gene expression 5. Mechanical Properties	Calcium deposition in the constructs	1 MPa	>90%	Solid Cuboidal	5 × 5 × 2 mm^3^	
**Yang et al., 2020****^31^**	GelMA and HAMA and LAP / type I collagen.	IDG-SW3 (Osteocyte cell line)	….	1x10^7^ cells per ml	***In-vitro*** 1.Osteocyte-related genes 2.Markers of osteocyte morphology 3.Markers of mineralization 4.Cellular response to parathyroid hormone (PTH) 5.Compression assay.	Osteocyte maturation and mineralization under 3D culture conditions.	38±2 kPa	85–90%	1.Pyramid structure 2.lattice structure	1.2 cm∗1.2 cm∗1.2 cm 10 mm∗10 mm∗2.4 mm	
**Kuss et al., 2017****^30^**	MeHA + GelMA + HA	SVFC	….	4 x10^6^ cells/mL	***In-vitro*** 1. Cell viability. 2. Vascularization 3.Osteogenic differentiation. ***In-vivo*** Subcutaneous implantation in Eight-week old, female athymic nude mice 1. Vascularization capacity.	Vascularization And Osteogenic differentiation	52 MP	----	Disc Shaped	8mm x1mm	
**Kang et al., 2016**^**29**^	Gelatin + fibrinogen + HA + glycerol	hAFSCs	....	5 x10^6^ cells/mL	***In-vitro*** 1. Cell viability 2. Cell proliferation 3. Osteogenic differentiation ***In-vivo*** 1. Modified Tetrachrome staining for Bone regeneration. 2. Von Willebrand factor immunostaining for large blood vessel formation	Human mandible bone	….	91± 2% (day 1)	Arbitrary shape	3.6 cm × 3.0 cm × 1.6 cm	
				3 × 10^6^ cells/ml		Calvarial Bone in rats		97 ± 6% (day 1	Circular shape	8 mm diameter × 1.2 mm thickness	

**Abbreviations**: nano-hydroxyapatite (particles) (nHAp), platelet-derived growth factor-B encoded plasmid-DNA (pPDGF-B), pDNA encoded with bone morphogenetic protein-2 (pBMP2)), Primary rat bone marrow stem cells (rBMSCs), Bone volume (BV),Total volume (TV), Bone mineral density (BMD), (β-TCP) β-tricalcium phosphate, Human-Dental pulp stem cells (hDPSCs), recombinant human bone morphogenetic protein-2 (rhBMP-2), Micro-computed tomography (μCT), Human periodontal ligament stem cells (hPDLSCs), ALP activity- Alkaline Phosphatase Activity, Gelatin Methacrylamide (GelMA), kappa carrageenan(kCA), Nanosilicates (nSi), Nanoengineered ionic covalent entanglement (NICE),Bone marrow derived human mesenchymal stem cells (hMSCs),Extracellular matrix (ECM), Amorphous magnesium phosphates, Bone Density(BD), Dentin sialophosphoprotein (DSPP),Osteocalcin (OCN), hyaluronic acid methacrylate (HAMA), Phenyl-2,4,6-trimethylbenzoylphosphinate (LAP), Methacrylated HA (Me-HA), Methacrylated Gel (Me-Gel), Hydroxyapatite (HA), Stromal vascular fraction derived cells (SVFC), human amniotic fluid–derived stem cells (hAFSCs).

## 3 Three-dimensional bioprinting for regeneration of periodontal tissues

The human periodontium, responsible for anchoring teeth, comprises of both hard and soft tissues. Being a part of the tooth-alveolar bone complex, its developmental progression involves dentinogenesis, cementogenesis, and osteogenesis. The initiation of periodontium formation begins with the inception of root development.After the crown formation concludes, the histomorphogenesis of the root proceeds with the downward extension of HERS (Hertwig's Epithelial Root Sheath). This prompts dental papilla cells to transform into odontoblasts, responsible for the generation of root dentin. As root dentin forms, the continuity of HERS is disrupted, permitting the inner layer cells of the dental follicle to come into contact with the already developed root dentin. This interaction leads to the transformation of these cells into cementoblasts. Simultaneously, the cells from the outer dental follicle layer differentiate into fibroblasts and osteoblasts, which collectively contribute to periodontal tissue formation. Several genes and signaling molecules coordinate the different phases of periodontium formation. For example, SHH/MSX-2 and IGF-1/BMP-4 interact to regulate HERS formation. HERS elongation and enhanced cell proliferation in its outer layer are caused by activation of IGF-1 receptors. The nuclear factor I family member NFIC is an essential transcription factor that controls the development of root odontoblasts. Furthermore, BMP-2 and BMP-7, two growth factors produced by HERS cells stimulates the production of CP23, a cementoblast marker. Members of the BMP family generally encourage bone formation and play a critical role in bone development. Inflammatory and non-inflammatory periodontal conditions can lead to tissue damage and potential tooth loss if untreated.^4^ When the periodontal structure is harmed, its natural regenerative capacity is limited and depends on the presence of MSCs at the defect site. Successful regeneration involves recruiting locally sourced progenitor cells that differentiate into cementum, PDL, and bone-forming cells. Regenerating a single tissue is a challenging task, and when it comes to periodontal regeneration, which involves three different tissue types, the complexity increases. It becomes even more formidable owing to the complex hierarchical architecture comprising of a soft tissue interspersed between two distinct hard tissue structures.^38^ Also goal of periodontal therapy is not just to regenerate tissues but also to restore the original structure and function of periodontal tissues.^39^ To achieve this, simultaneous formation of the bone-PDL-cementum complex should occur with osteogenesis, slightly preceding cementum and PDL fiber differentiation.^40^ Then proper attachment and orientation of PDL fibers to the newly formed cementum and alveolar bone should follow this process. The concurrent restoration of structures supporting a lost tooth relies significantly on the interaction among scaffold, cells, and bioactive signals. The introduction of the multilayered/multiphasic scaffold strategy is making it increasingly viable to achieve simultaneous regeneration of two or more tissues, yielding successful results Multiphasic scaffolds find application in various tissue engineering contexts, such as bone, cartilage, and periodontal tissues. Techniques involving double/bilayered, biphasic, hybrid scaffolds, and similar constructs have been employed for the regeneration of periodontal tissues. Bioinks deemed appropriate for building multi-layered or multiphasic scaffolds for PDL regeneration encompass both natural^41^ and synthetic materials.^42^^,^^43^ (Table 2) Artificial polymers are manufactured using inorganic sources and are categorized into absorbable and nonabsorbable polymers. Resorbable polyesters, such as PCL, polylactic acid (PLA), polyglycolic acid (PGA), polylactic-polyglycolic acid (PLGA), PEG, and PEG with PLGA (PEG-PLGA), are prevalent within synthetic polymers. Among these, PCL stands out as the most representative synthetic polymer. To address the intricate structural challenges, Park et al.,^44^ utilized a Solidscape PCL, a linear synthetic bioresorbable aliphatic polyester approved by the FDA (Food and Drug Administration), exhibits remarkable thermal stability and the ability to take on diverse forms, distinguishing it from other materials employed in tissue engineering scaffolds.^45^ Its hydrophobic nature allows it to impede medium access and regulate drug dissolution. However, this hydrophobic characteristic poses challenges for cell attachment, proliferation, and differentiation.^46^ Consequently, surface modifications are essential. The ease of processing PCL has led to its utilization in regenerating various tissue defects through three-dimensional (3D) printing. A calcium phosphate (CaP)-coated polycaprolactone (PCL) was created by Dan et al.,^47^ to implant cell sheets into exposed root surfaces in an athymic rat model having periodontal defects. Thermosensitive poly (N-isopropylacrylamide) (PiPAam) dishes were used to culture Human periodontal ligament cells After that, three layers of cell sheets were placed on CaP-PCL and applied to the defect site. After four weeks of implantation,the results demonstrated that the inclusion of cell sheets encouraged the creation of the PDL, whereas CaP-PCL alone facilitated bone formation. Furthermore, a multiphasic scaffold made of PCL-HA (90:10 wt%) with interlaid strands (diameter, 100 μm) was created by Lee et al.,^48^ using an extrusion-based bioprinter. It had three phases namely phase A, phase B, and phase C, where these strands created interconnected microchannels with widths of 100, 600, and 300 μm, respectively. Additionally, phases A, B, and C were coated with PLGA microspheres containing BMP-2, connective tissue growth factor (CTGF), and recombinant human amelogenin, respectively. On subcutaneous implantation in immunodeficient mice and 4 weeks of culture in a specified medium revealed that hDPSCs implanted on this scaffold underwent dentin/cementum, PDL, and alveolar bone differentiation. To address the intricate structural challenges, Park et al.,^42^ utilized a Solidscape printer Model maker II (extrusion printing) for 3D printing of a wax mold. Subsequently, they employed an extrusion-based bioprinter to create biomimetic hybrid scaffolds featuring distinct polymer regions such as the PCL compartment for bone regeneration and the PGA compartment for PDL formation. The PCL and PGA compartments were seeded with primary human gingival fibroblasts (hGF) and human periodontal ligament cells (hPDLCs) in a fibrinogen solution, respectively. Human dentin slices with exposed dentinal tubules were employed to cap the structures after thrombin was used to achieve gelation. After that, the complete constructions were placed subcutaneously into an immunodeficient mouse model's dorsa and left there for six weeks. The results showed how ligament and bone tissues could be created with precise localization and organization. In order to accomplish complete and concurrent regeneration of cementum, alveolar bone, and PDL at a periodontal defect site, Sowmya et al.,^49^ employed a three-layered scaffolding technique. Cementum Protein 1 (CEMP-1), recombinant human fibroblast growth factor (rhFGF), and platelet-rich plasma (PRP) were loaded into a porous three-layered hydrogel scaffold composed of chitin-poly(lactic-co-glycolic acid) (PLGA)/nanobioactive glass ceramic (nBGC), which was lyophilized in each of the three layers. Microcomputed tomography analysis showed that complete closure of the defects and healing with the formation of new cancellous-like tissue occurred after implantation, either with or without growth factors, into rabbit maxillary periodontal defects. The results were compared with those of the control group at 1 and 3 months postoperatively.

**Table 2:** Design Features of Studies Utilizing 3-D Bioprinting for regenerating Periodontal tissues.

*References*	*Bio-ink*			*Cell Density*	*Scaffold Model*	*Assessments*	*Tissue Regeneration*	*Dimension*	*Bio-printed Construct*
	Scaffold Material	Cell Seed	Bioactive Factor(BF)						
Sowmya et al., 2017^49^	Chitin-PLGA/.nBGC	hDFCs	Cementum Layer: Cementum protein 1 PDL Layer:Fibroblast growth factor 2 Alveolar bone layer: Platelet-rich plasma derived growth factors	20 000 cells/cm^2^	Tri- Layered	**In-vitro** 1.Protein Expression-COL1, CEMP1, and BSP, FSP, PLAP1, RUNX2 and human OCN 2. Alkaline Phosphatase Activity 3. Biomineralization by the Cellular Method **In-Vivo** Scaffold was assessed in Rabbit's Maxillary Periodontal defect – 1Micro-CT Analysis 2. Histological Analysis	New Cementum, Fibrous PDL, and Alveolar bone	6 × 5 × 1 mm^3^	
Lee et al., 2014^48^	PCL and HA	Phase A: DPSCs Phase B: PDLSCs Phase C:ABSCs	Phase A: Amelogenin Phase B: CTGF Phase C: BMP-2	1 × 10^5^ cells/scaffold	Tri-phasic	**In-Vitro** Multiphase tissue formation in cell-seeded scaffolds **In-Vivo** Multiphase scaffolds were implanted in subcutaneous pouches of 10-week-old immunodeficient mice (Harlan)	Cementum, PDL and Alveolar like tissue	5 × 5 × 3mm^3^	
Dan et al., 2014^47^	CaP-PCL	hPDLC, hGMC and hABC	….	For 1. Von Kossa staining- 1 × 10^4^ cells/well 2. Hydroxyapatite formation- 1 × 10^4^ cells/well 3. Alizarin Red S staining-2 x10^4^ cells/well 4. BSP gene expression- 1 × 10^5^ cells/well	Bi-layered	**In- Vitro** The osteogenic potential of the three cell types was initially assessed by measuring both mineralization (Von Kossa staining) and hydroxyapatite formation and Alazarin Red S staining and assessment of BSP mRNA expression. **In-vivo** A surgical defect was created in the mandibular first molar area of 12-week-old athymic rats and were assessed for New Bone, Cementum and PDL fomation by 1.Histomorphometric analysis. 2.Immunohistochemistry	New Cementum. New Alveloar Bone and New PDL	**CaP-PCL scaffold** 3 mm × 1.5 mm x 0.5 mm **PCL Membranre**- 8 mm × 5 mm x 0.3 mm	Ca-PCLScaffold PCL Membrane
Park et al., 2012^42^	PCL solution in acetone	hPDLC	AdCMV-BMP-7	1. hPDL −2.4 × 10^5^ cells/scaffold 2. Ad-BMP-7-hPDL −2.4 × 10^5^ cells/scaffold	Bi-phasic	**In-vivo** Bioprinted scaffold was placed in surgically created periodontal osseous (fenestration defects) on the buccal side of the distal root surface of mandibular 1st molar tooth in Athymic rats 1.BVF 2.TMD 3. Histomorphometric analysis 4 Immunofluorescence staining	1.Alveolar bone formation. 2.Fiber formation and orientation in the ligamentous portion of the scaffold	….	….
Park et al., 2010^44^	PCL-PGA	Bone Region:Ad-BMP-7-hGF cells PDL interface: hPDL cells	AdCMV-BMP-7	Bone region: 2.4 × 10^5^ cells PDL Interface: 1.4 × 10^5^ cells	Bi-Phasic	**In-Vivo** 3D –Bio-printed Implant was placed in surgically created subcutaneous pockets of the nude mouse 1.BVF 2. BMD 3. Percentage Length of cementum-like tissue formation	Alveolar Bone, Cementum and PDL	**Bone region**:0.75 × 0.50 × 0.05 mm^3^**PDL Interface**: 3.5 × 4.5 × 0.8 mm^3^	

**Abbreviations:** Poly(lactic-*co*-glycolic acid) (PLGA); Nano-bioactive glass ceramic (nBGC); Human Dental Follicle Cells (hDFCs); Collagen type 1 (COL1); Cementum Protein 1(CEMP1); Bone sialoprotein (BSP) protein; Fibroblast surface protein (FSP); Periodontal ligament associated protein 1 (PLAP1); antihuman runt-related transcription factor 2 (RUNX2); antihuman osteocalcin (OCN); Polycarprolactione (PCL),Hydroxylapatite (HA), Dental pulp stem/progenitor cells (DPSCs); Periodontal Ligament stem/progenitor cells (PDLSCs); CTGF (Connective Tissue Growth Factor); Alveolar bone stem/progenitor cells (ABSCs); Bone Morphogenic Protein (BMP-2); Calcium phosphate -polycaprolactone (CaP-PCL); Human periodontal ligament cells (hPDLC); Human Gingival margin-derived cells (hGMC); Human Alveolar bone-derived cells (hABC); recombinant adenovirus-encoding murine bone morphogenetic protein-7 (AdCMV-BMP-7); Bone Volume Fraction (BVF); Tissue Mineral Density (TMD); Poly(glycolic acid) (PGA); adenovirus encoding BMP-7 transduced human gingival fibroblast cells (Ad-BMP-7-hGF).

## 4 Three-dimensional bio-printing for the regeneration of dental pulp

The dental pulp is a highly vascularized and a well innervated connective tissue located in the middle of a semi-permeable structure made up of dentin tubules and a mineralized matrix. This tissue spans from the root apex to the crown and occupies a chamber with a long canal. The specialized cells known as odontoblasts are located in a pseudo-stratified layer along the edge of the pulp chamber and root canal. They are essential to the development of dentin, the tissue that surrounds the pulp. In the pulpal tissue, in addition to fibroblasts, neurons, and resident stem cells, there is a network of blood capillaries that run centrally through the pulp and out toward the tooth crown. A capillary-rich plexus that is situated a few micrometers away from the odontoblast layer close to the dentin is created by microcapillaries that branch forth from the core vessel.^50^ In case of profound caries or tooth trauma, the equilibrium of pulp tissue is disrupted, requiring the need for root canal therapy to preserve the tooth. Thus the concept of regenerative endodontics which aims to regenerate pulp tissue has been suggested as an alternative to traditional root canal procedures to restore tooth function. Dental Pulp regeneration like any other tissue regeneration relies on three crucial elements: scaffolds, cells and growth factors.^51^^,^^52^ Similarly successful regeneration of dental pulp also involves the stimulation of a capillary network and the creation of a conducive environment within a confined space. Essential aspects for accomplishing this include the careful choice of the necessary growth factor, an effective delivery system for the growth factor, and a scaffold that induces the development of blood vessels and stem cells from the residual pulp. These factors are vital in establishing local regeneration of the dentin–pulp complex. Scaffolds intended for regenerative purposes in dental pulp must replicate the microenvironment found in the root canal while offering mechanical support. Therefore, they need to be optimized both in terms of timing and spatial configuration to facilitate efficient cell attachment and proliferation.^33^ Within dentin–pulp complexes, hDPSCs function as mesenchymal stem cells, situated at the core of the pulp tissue. The outer region comprises dentin tissue, essentially a mineralized ECM containing a differentiated odontoblastic layer. Therefore, it is crucial to regulate this spatial aspect of odontogenic differentiation in hDPSCs within the three-dimensional dentin–pulp complex.^33^ It is also necessary to create a conduit that functions as a blood vessel within the designed pulp scaffolds from the moment the regenerative process begins. Later on during the remodeling process, this will maximize the diffusion of nutrients and oxygen and make waste elimination easier over the scaffolds' whole length. Furthermore, the root apex-to-microchannel pathway is meant to facilitate host cell migration and integration into the scaffold structure. It is imperative to highlight that complete and effective dental pulp regeneration necessitates not only the engineering of the pulp vasculature but also the manipulation of all cellular and tissue constituents that make up the tissue structure, such as immune cells, fibroblasts, nerves, and stem cells.^53^ Researchers^33^^,^^54^^,^^55^ have endeavored to rejuvenate pulpal tissue by showcasing the transformation of stem cells loaded onto a 3-D bio-construct into odontoblasts triggering the initiation of dentine matrix formation and identifying markers/proteins linked to the mineralization process. Furthermore, some researchers^53^^,^^56^ have explored pulpal regeneration by illustrating the creation of vascularized tissue as a component of regenerative endodontics, with the goal of restoring the vitality of tooth(Table 3). Numerous bioinks have been investigated for the 3-D bioprinting of scaffolds for the dental pulp tissue, each possessing several unique characteristics. Here are the different types of bio-inks and their scaffolds with distinct characteristics employed in the 3D bioprinting of dental pulp tissue.

**Table 3:** Design Features of Studies Utilizing 3-D Bioprinting for regenerating Dental pulp.

*References*	*Bio-ink*			*Cell Density*	*Assessments*	*Tissue Regeneration*	*Cell Viability*	*Shape*	*Dimension*	*Bio-printed Construct*
	Scaffold Material	Cell Seed	Bioactive Factor(BF)							
Choi et al., 2022^55^	GelMA-MTA GelMA	hDPSCs	….	5 × 10^4^ cells per well	**In-vitro** 1.Cell Adhesion. 2.Cell Viabillity 3. Odontogenic Differentiation and Mineralization	Dentin/pulp Tissue.	….	… .	….	
Duarte Campos et al., 2019^56^	Agarose and collagen type I	hDPSCs/HUVECs	… .	hDPSCs -3x10^6^ cells per ml HUVECs- 3 × 10^6^ cells per ml	**Ex-vivo** 1. Oscillatory rheological analysis. 2.Immunocytochemical staining and vasculogenesis Analysis.	Dental Pulp	….	Bovine Root Shaped	Length- 8 mm Diameter-4-5 mm	Root Canal Prepared Bovine Root
Yu et al., 2019^54^	Alg-Gel	hDPSCs	….	….	**In-vitro** 1.Cell Growth 2. Cell Adhesion 3.Osteogenic and odontoblastic differentiation of cells 4. Expression of Mineralization associated genes	Dental Pulp	….	Round	10 mm in diameter	
Han et al., 2018^33^	Fibrinogen/gelatin/hyaluronic acid/glycerol	hDPSCs	….	3 × 10^6^ cells/mL	**In-vitro** 1.Cytocompatibility 2. Odontogenic differentiation	Dentin -Pulp complex	>90 %	Human Tooth shaped	8 mm × 8 mm × 20 mm	
Athirasala et al.,2017^53^	GelMA	OD21	….	5 × 10^6^ cells/mL	**In-vitro** 1. Cell viability 2.Cell spreading 3.Cell proliferation 4.Formation of endothelial monolayers by ECFCs.	Pre-vascularized pulp-like tissue	>90 %	Root fragment of Human pre-molars	Height- 9 mm Diameter- 1.5 mm	

**Abbreviations**: Gelatin Methacrylated -Mineral trioxide aggregate (GelMa -MTA); human dental pulp stem cells (hDPSCs); Human primary umbilical vein endothelial cells (HUVECs); Alginate/Gelatin (Alg-Gel); Gelatin methacryloyl (GelMA); Odontoblast-like cells (OD21); Endothelial colony forming cells (ECFCs).

### 4.1 Alginate-based bio-inks

Tissue engineering has made use of the alginate/gelatin hydrogel (Alg-Gel) scaffold.Yu et al.,^54^ in 2019 cultured hDPSCs on a scaffold made of this hydrogel to examine its effects on cell proliferation, adhesion, and the expression of genes involved in mineralization. According to the results, 3D printed Alg-Gel was more favorable for hDPSC growth than regular Alg-Gel, and scaffold extracts can therefore more effectively promote cell proliferation and differentiation. In order to potentially aid in the future regeneration of the dentin-pulp complex for tooth repair, this study lays the theoretical groundwork for the biological properties of a 3D bioprinted alginate/gelatin hydrogel scaffold.

### 4.2 Gelatin Methacrylate (GelMA) based bio-inks

Also, in 2017, Athirasala et al.,^53^ encased odontoblasts (OD21) in a GelMA 15 % hydrogel with a centrally designed channel made using the sacrificial template technique. Following this, they implanted the construct into the human teeth's roots by introducing endothelial colonyforming cells (ECFCs) into the channel. ECFCs formed a monolayer after being incubated for 7days in DMEM-EGM-2MV (1:1) media. Throughout the GelMA hydrogel, angiogenic sprouting was seen, mimicking pulp-like tissue. GelMA and GelMA-MTA were used by Choi and colleagues^55^ in 2022 to make 3D scaffolds using an extrusion-based 3D bioprinter. The proliferation and differentiation of primary hDPSCs were then evaluated by seeding them onto these scaffolds. The researchers noticed increased odontogenic differentiation after a 7-day culture in differentiation medium. This was demonstrated by elevated levels of Alkaline phosphatase (ALP) and the expression of genes for dentin matrix acidic phosphoprotein 1 (DMP-1) and dentin sialophosphoprotein (DSPP), especially in the GelMA-MTA bioprinted constructs.

### 4.3 Fibrin/hyaluronic acid-based bio-inks

Within the dental pulp complex, hDPSCs are situated at the center of the teeth, while dentin is found in the outer region. To explore the potential for spatially regulating hDPSC differentiation, Han and colleagues ^33^ in 2019,employed fibrin-based bioinks (comprising fibrinogen, gelatin, hyaluronic acid, and glycerol) with varying concentrations of fibrinogen (5, 10, 15, 20 mg/mL) to control stiffness spatially. Using a custom bioprinter utilizing extrusion bioprinting and based on a dental shape captured by computed tomography (CT), hDPSCs were bioprinted. Following 15 days of culture in differentiation medium, the outcomes revealed spatial odontogenic differentiation, with the central pulp region remaining undifferentiated, while there was localized deposition of dentin in the outer region.

### 4.4 Collagen-based bio-inks

Additionally, Duarte Campos and associates^56^ in 2020 studied co-cultures of primary human umbilical vein endothelial cells (HUVECs) and hDPSCs encapsulated in different bioinks (0.2 % collagen type I-0.5 % agarose, 0.5 % fibrin, and 0.3 % collagen type I) for the regeneration of dental pulp tissue. Using a handheld bioprinter, these bioinks were introduced inside the root canals of bovine teeth. Following a 14-day culture period, the researchers observed the development of new capillary networks in all tested samples.

## 5 Three-dimensional bio-printing for TMJ regeneration

The temporomandibular joint (TMJ) acts as the junction connecting the glenoid fossa, the articular eminence of the temporal bone, and the mandibular condyle, incorporating a fibrocartilaginous disc in between. Problems involving the TMJ stand out as the primary cause of enduring or recurrent orofacial pain. Temporomandibular disorders (TMD) encompass a wide range of pathologies related to the muscles of mastication, the TMJ and the surrounding structures of bone and soft tissues.^57^ Among TMD, TMJ osteoarthritis which is considered to be a degenerative condition of a lowgrade inflammation, affecting either one or both the temporomandibular joints, is prevelant among 9.8 % of the adult and elderly populations.^58^ Among the intricate contributing factors to TMJ osteoarthritis, the primary instigator is thought to be heightened mechanical stress on the articular fibrocartilage whether it is in a healthy or compromised state. Increased stress may arise from conditions such as mandibular asymmetry, severe malocclusion, and excessive muscular strain. Locally, this elevated mechanical load can lead to both the displacement and deterioration of the TMJ disc, ultimately resulting in the progressive development of osteochondral defects in the mandibular condyle.^59^ Hence, TMJ osteoarthritis causes damage to the fibrocartilage within the TMJ disc and mandibular condyle. This damage leads to localized pain and diminished functionality, ultimately impacting the overall quality of life of the affected patients. Thus utilization of 3-D bioprinting in tissue engineering presents a promising pathway for potentially regenerating fibrocartilage in the TMJ disc and mandibular condyle, offering a viable and innovative treatment options for patients with OA. In the field of cartilage tissue engineering, a considerable amount of research has focused on exploring natural biomaterials substances like collagen, silk fibroin, fibrin, gelatin, sodium alginate (SA), hydroxyapatite, HA, agarose, chitosan, chondroitin sulfate, and decellularized extracellular matrix (dECM). Despite their commendable biocompatibility and cytocompatibility, these natural biomaterials inherently exhibit certain drawbacks, such as inadequate mechanical properties, limited thermal stability, and inappropriate degradation rates. In contrast, synthetic biomaterials like polyvinyl alcohol (PVA), PEG, PCL, polyurethane (PU), PGA, PLGA, and PLA have gained increasing prominence. Synthetic biomaterials offer advantages such as enhanced printability, structural stability, and controlled mechanical properties and degradation rates compared with their natural counterparts. Nevertheless, some undesirable properties, such as bioinertness and slow degradation rates in synthetic materials, pose challenges for articular cartilage regeneration. Recent endeavors have sought to address these challenges by combining natural and synthetic biomaterials, which has led to the development of hybrid scaffolds. This innovative approach aims to capitalize on the strengths of both natural and synthetic materials, providing an effective strategy to enhance overall scaffold performance. Thus researchers have implemented various strategies, including the integration of suitable scaffold materials—whether natural, synthetic, or a combination of both—along with seeded cells and bioactive factors (BF)(Table 4).

**Table 4:** Design Features of Studies Utilizing 3-D Bioprinting for regenerating TMJ.

*References*	*Bio-ink*			*Cell Density*	*Assessments*	*Tissue Regeneration*	*Shape*	*Dimension*	*Bio-printed Construct*
	Scaffold Material	Cell Seed	Bioactive Factor(BF)						
Jiang et al., 2021^60^	(1) PCL + PVA (2) PVA	Rabbit chondrocytes and fibroblasts	….	….	**In-vitro** 1.Mechanical Behaviors 2.Tribological Characteristics Surface 3.Morphologic Characterization 4.Cell Proliferation and Adhesion **In-Vivo** PVA + PCL disc was implanted into the lateral third of the TMJ disc in the male goats to mend the defect, directly covering the condylar cartilage surface. 1.Histological analysis.	TMJ Disc	….	….	
Moura et al., 2020^61^	PCL + PEGDA	….	….	….	**In-vitro** Influence of 1.Processing temperatures, 2.Filament diameter 3. Biological environment.	TMJ disc	Geometry corresponding to the native sheep TMJ Disc	….	
Legemate et al., 2016^62^	PLGA μS + PCL	Human BMSCs	CTGF, TGF-β3	2 × 10^6^ cells/mL	**In-vitro Mechanical Tests** 1.Compression Test 2.Tensile Test 3.Stress relaxation tests	TMJ Disc	Disc-shaped samples	5 × 2 mm^2^	
Abramowicz et al.,2021^65^	PCL	….	BMP-2	….	**In-Vivo** Initially, the scaffold was implanted in the temporalis muscle to promote vascularity. Six weeks later, a unilateral condylectomy was performed, and the vascularized scaffold was rotated onto the defect. After six months, the pigs were sacrificed, and assessments were conducted individually for clinical, mechanical, radiographic, and histologic findings.	Pediatric TMJ mandibular condyle Reconstruction	Mini-pig-specific mandibular condyle shaped scaffold	….	
Wang et al., 2017^64^	PCL/HA PGA/PLA	Minipig BMSCs Minipig chondrocytes	5 × 10^7^ cells/mL 2.5 × 10^7^ cells/mL	…. ….	**In- vitro** Microstructure and Cell adhesion using SEM **In -vivo** Scaffolds were subcutaneously implanted in nude mice. Following a 12-week period the specimens were collected for 1Histopathological analysis using hematoxylin and eosin, safranin O-fast green staining. 2.Immunohistochemical staining for collagen I and II.	Regeneration of condyle-shaped osteochondral composite.	Anatomic size of mandibular condyle of the minipig.	13 x 11 × 10 mm^3^	Bone-phase scaffold Cartilage phase scaffold
Schek et al., 2005^63^	HA/PLA	Transduced HGFs and Pig chondrocytes	AdCMV-BMP-7	5 × 10^7^ cells/mL	**In- vivo** Subcutaneously implantation of bi-phasic scaffold into mice. 1.Histological Analysis.	Osteochondral tissue regeneration	Cylindrical	Height-3mm Diameter-5mm	Upper polymer phase (white) and the lower Ceramic phase (blue)

**Abbreviations:** poly(D, L-lactic-co-glycolic acid) (PLGA); microspheres (μS); polycaprolactone (PCL); BMSCs, Bone mesenchymal stem cells; Connective tissue growth factor (CTGF); transforming growth factor beta 1(TGF-β1); poly(ethylene glycol) diacrylate (PEGDA); polyvinyl alcohol (PVA); Hydroxyapatite (HA); human gingival fibroblasts (HGFs); a recombinant adenovirus construct expressing the murine BMP-7 gene under a cytomegalovirus promoter (AdCMV-BMP-7); polyglycolic acid/polylactic acid (PGA/PLA).

### 5.1 3-D printed scaffolds for TMJ disc cartilage tissue regeneration

Several strategies have been suggested to achieve the spatial and temporal release of different growth factors within 3D printed scaffolds loaded with mesenchymal stem cells (MSCs), considering TMJ disc as a fibrocartilage structure comprising fibroblasts and chondrocytes. In a technique outlined by Jiang et al.,^60^ in 2021 for modifying the PCL scaffold, the process involved immersing the 3D-printed PCL scaffold in a PVA hydrogel solution which resulted in cross-linked network through three freeze-thaw cycles. Through in-vitro tests, the PVA-PCL scaffold was shown to be non-cell adhering, to have a smooth surface, and to have similar porosity, and compressive and viscoelastic qualities similar to those of a natural TMJ disc. After that, the PVA-PCL scaffold was surgically inserted into goats to replace the lateral third of the disc. Following three months, histological examination showed visible fibrous tissue ingrowth surrounding the PCL but no degenerative alterations in the mandibular condyle. This phenomenon could be attributed to the gaps between PVA and PCL, providing spaces conducive to fibrous tissue ingrowth, thus presenting a potential advantage of the PVA-PCL scaffold. In their 2020 study, Moura and colleagues^61^ utilized PCL and poly(ethylene glycol) diacrylate (PEGDA) as materials for scaffolds in the bio-printing of TMJ discs resembling those of black Merino sheep. The in-vitro evaluation of the bio-printed TMJ disc focused on factors affecting mechanical properties, such as processing temperatures, filament diameter, and the biological environment. The study also examined two multimaterial approaches, incorporating a hydrogel shell or a hydrogel core into the scaffold. The results revealed that an increase in temperature led to decrease scaffold porosity and increase compressive modulus. A reduction in filament size (from 300 to 200 mm) resulted in nearly halving the compressive modulus compared to the initial value, with 200 mm filaments exhibiting a modulus closer to that of the native tissue. Introducing a hydrogel core improved the mechanical properties of the TMJ disc substitution. In 2016, Legemate and associates^62^ identified TGF-β3 as a chondrogenic factor and CTGF (Connective Tissue Growth factor) as a pro-fibrogenic factor, encasing both within PLGA microspheres (μS). Two variants of PLGA μS were then included (using an EBP) into 3Dprinted PCL scaffolds to allow for the temporal and spatial release of growth factors (GFs). Fluorescence imaging revealed that TGF-β3was primarily concentrated in the intermediate band of the scaffold, whereas CTGF was distributed throughout the entire area. After six weeks of MSC cultivation, the spatiotemporal delivery of GFs resulted in the creation of heterogeneous fibro cartilaginous tissue within the scaffold. Furthermore, scaffolds with a greater dose of GFs/μS showed larger amounts of collagen and GAG (Glycosaminoglycan) in the anterior/posterior bands and the intermediate zone when compared to scaffolds with a lower dose. However, the tensile modulus of the scaffolds was unaffected by the GF/μS dose.

### 5.2 3D-printed scaffolds for mandibular condyle cartilage tissue regeneration

A biphasic scaffold was made with PLA/HA in 2005 by Schek and colleagues^63^ through image-based design and indirect solid free form construction. This novel method entailed planting porcine knee joint chondrocytes into the upper polymeric phase and genetically altered human gingival fibroblasts—expressing BMP-7 by an adenovirus—into the bottom ceramic phase. The biphasic scaffold showed amazing success in repairing cartilage, bone, and an osteochondral interface in mice following a 4-week subcutaneous implantation. Nonetheless, the existence of tiny cartilage pockets inside the ceramic phase's pores highlighted the necessity of more control over the spatial distribution of regenerated tissue. In 2017, Wang and colleagues^64^ developed the PLA/HA biphasic scaffold further by integrating a PGA/PLA scaffold, seeded with chondrocytes, and a PCL/HA scaffold, seeded with bone MSCs. There were visible structural variations between the two phases of the resulting comprehensive biphasic scaffold. After a 12-week subcutaneous implantation into the dorsum of mice,a smooth, homogeneous, cartilage-like tissue, about 1.2 mm thick, covered the surface of the scaffold. Histological analysis revealed the interface between the regenerated cartilage and the subchondral bone and validated the presence of regenerated cartilage. Interestingly, given that bone mesenchymal stem cells (BMSCs) can differentiate into two distinct phases, the addition of auricular chondrocytes to the PGA/PLA scaffold may have attenuated the production of bone within the microchannels of the PCL/HA scaffolds. Therefore, more investigation is necessary to clarify the impact of chondrocytes (auricular and articular) on BMSCs and to maximize the creation of the osteochondral interface. In 2021, Abramowicz et al.,^65^ continued the trajectory of advancements by utilizing a customized 3D-printed scaffold coated with BMP-2 for reconstructing the mandibular condyle in pediatric TMJ cases in porcine subjects. Employing a pedicled (temporal) flap during the implantation procedure, they conducted CT scans after a 6-month period to compare the reconstructed condyles with the unoperated (control) TMJ. The findings showed that the 3Dprinted polycaprolactone TMJ incorporating BMP-2 maintained its overall shape, mandibular height, condylar volume, and bone volume fraction when compared to the control group.

## 6 Three-dimensional bioprinting for the regeneration of the whole tooth

A tooth is a complex structure comprising enamel, dentine, cementum, and dental pulp, and it relies on the support of the surrounding periodontal tissues. Following damage, a tooth's capacity for self-repair is limited. Present clinical interventions to address complete tooth loss involve the use of dentures or dental implants. Recent progress in stem cell research, biomaterial development, and tissue engineering has spurred numerous research initiatives aimed at revolutionizing techniques for engineering teeth and achieving complete regeneration. In the past, medical professionals have explored the regeneration of artificial teeth using cellbased therapies.^66^ In these endeavors, tooth germs were often introduced into various animal models to examine subsequent tooth formation.^67^ Throughout the process of tooth development, individual tooth germs progress through distinct stages, known as the bud, cap, and bell stages, respectively, each marked by unique morphological changes. Vital signaling pathways such as BMP, FGF, and TGF-β, along with pivotal transcription factors like Wnt and Shh, hold significant and determinative roles at different stages, ensuring the successful progression of tooth development. These factors and genes are indispensable for guiding tooth germ morphogenesis from the bud to the bell stages, orchestrating the differentiation of odontoblasts and ameloblasts during dentinogenesis and amelogenesis, overseeing the initiation and cessation of the mineralization process, and facilitating the accurate formation and elongation of HERS during root development. Any deficiency or distortion in these factors could severely impedes the intricate process of tooth formation. Scaffold-based methods were also introduced to stimulate the artificial regeneration of teeth, leading to the creation of tissue resembling tooth shapes.^68^ Nevertheless, both of these approaches fell short in creating customized composite tissues that include enamel, dentin, and pulp, tailored to individual patients.^69^ The main challenges associated with tooth germ-based techniques were the difficulty of controlling the size and shape of the regenerated tooth and the cultivation of primary biological sources for germ structures. Various bioinks have been investigated for tooth regeneration, encompassing both scaffold-based bioprinting technology and scaffold-based methods without bioprinting. Researchers have actively explored and formulated novel bioink compositions. In scaffold-based methods without bioprinting for whole tooth regeneration, commonly utilized bio-inks involve substances such as collagen and polymers such as PGA. In 2006, Iwatsuki et al.,^70^ employed 3D scaffolds measuring 1 x 1 × 1 mm, which were constructed from a PGA fiber mesh (with a diameter of 13 μm and a density of 60 mg/ml). Each scaffold was seeded with murine tooth germ cells at a concentration of 1 × 10^5^ cells per scaffold. Subsequently, these seeded scaffolds were surgically implanted into the kidney capsule of syngeneic male DDY strain mice aged 5–6 weeks with an observation period of 4 weeks. A study comparing a collagen sponge and a PGA fiber mesh as three-dimensional scaffolds for whole tooth tissue engineering was carried out in 2006 by Sumita et al.,^71^ The results of the study showed that, when it comes to tooth tissue engineering, the collagen sponge scaffold performed better than the PGA fiber mesh scaffold. In 2007, Honda et al.,^67^ employed a collagen sponge scaffold, employing a technique where he in order to encourage direct interaction between the two cell types, mesenchymal cells were seeded onto the surface of the scaffold, followed by epithelial cells. In vitro and in vivo assessments of the resultant cell-scaffold structures were conducted in immune-compromised rats; control groups were set up to evaluate constructs in which direct contact between the two cell types was purposefully hindered. Scaffolds seeded using this innovative technique exhibited significantly higher alkaline phosphatase activity than the controls. Furthermore, the in-vivo tooth morphology closely resembled that of a natural tooth with each scaffold forming a singular tooth structure. Overall, scaffold-based approaches struggled to regenerate composite tissues with precision due owing to their inability to accurately position multiple cell types in predetermined arrangements. To surmount these limitations, researchers are advocating for the implementation of 3D bioprinting technology, which offers a unique means of constructing 3D cellular structures incorporating a variety of living cells, biomolecules, and biomaterials for the purpose of regenerating artificial teeth.^72^^,^^73^ This technology employed polymeric-based bio-inks, utilizing synthetic polymers like PCL and PLGA for tooth regeneration(Table 5). Kim et al.,^74^ made an initial effort towards whole tooth regeneration by employing an Extrusion-based bioprinter. They created a Human Mandibular molar-shaped and Rat incisor shaped scaffold using PCL (80 %)/hydroxyapatite (20 %) bioink, devoid of cells. In order to develop multiple tissues, a cell-homing strategy was implemented where researchers coated the scaffold with a mixture of stromal derived factor-1 (SDF1, 100 ng/mL) and bone morphogenic protein 7 (BMP-7, 100 ng/mL) in collagen type I solution. Subsequently, these scaffolds were implanted orthotopically in the extracted socket and ectopically in the dorsum in rats for a duration of 9 weeks. The outcomes revealed the formation of new alveolar bone, mineralization, the establishment of PDL and initiation of angiogenesis. An attempt was made to regenerate teeth using 3D printed hydroxyapatite/polylactic acid scaffolds infused with canine dental pulp stem cells in a follow-up study by Chen et al.,^75^ in 2021. In this each dog's left lower jaw received these scaffolds from the researchers, while the control group, that is, the dog's right jaw, received scaffolds devoid of cells. Sections of the lower jaw were taken nine months after the surgery and stained with hematoxylin and eosin for histological analysis. The results showed that, notably higher degree of mineralization was observed in the experimental group, where the scaffolds were seeded with cells, compared to the control group that received cell-free scaffolds. Recently, Gong et al.,^76^ (2022) utilized 3D bioprinted tooth-shaped scaffolds constructed from a composite bio-ink consisting of nacre, polyurethane (PU), and polyhedral oligomeric silsesquioxane (POSS) [NPP] for alveolar ridge preservation following tooth extraction. The NPP scaffolds were evenly seeded with the MC3T3-E1 cells. In-vitro assessments were conducted to analyze the rheological behavior, mechanical properties, and biomineralization. For in-vivo evaluation, the NPP scaffolds were implanted into the sockets following the extraction of mandibular incisors. This resulted in reduced alveolar bone resorption in terms of both height and width, accompanied by observable new bone formation.

**Table 5:** Design Features of Studies Utilizing 3-D Bioprinting for regenerating Whole Tooth.

*References*	*Bio-ink*			*Cell Density*	*Assessments*	*Tissue Regeneration*	*Shape*	*Dimension*	*Bio-printed Construct*
	Scaffold Material	Cell Seed	Bioactive Factor(BF)						
Gong,et al., 2022^76^	PU/(POSS)	MC3T3-E1 cells	Nacre	5 × 10^3^ cells per well	**In-vitro** Thermal characterization Water contact angles Surface roughness Rheology and printability analysis. Mechanical properties. Biomineralization Cell proliferation and live/dead assay. Cell osteogenic differentiation **In-Vivo** 1.Micro-CT evaluation: BMD and BVF 2.Measurement of alveolar bone resorption. 3.Histological analysis. 4.Toxicity in vivo.	Tooth shaped scaffolds for alveolar ridge preservation	Cuboid-shaped	10 x10 × 5 mm	Cuboid Shaped Bioprinted Scaffold Tooth shaped scaffolds for in-vivo implantation
**Chen et al.,2021**^75^	HA/PLA	dDPSCs	….	1 × 10^5^ cells/scaffold	**In-Vivo** Surgically created defects of known dimensions were implanted with HA/PLA + dDPSCs (Experimental side) and HA/PLA (Control side) scaffolds in the right and left side of male beagle dogs after 4 weeks post extraction healing of the bilateral premolars (P2 and P4) and third incisor (I3) 1.Cone Beam CT and Micro CT Observation	Feasibility assessment for the regeneration of incisor and premolar teeth	Cylindrical	Incisor Scaffold(I-scaffold) - 3 × 8 mm Premolar scaffold (P-scaffold)-6x 8 mm	Side View Top View
Kim et al., 2010^74^	Poly-Ʃ-caprolactone and hydroxyapatite	….	SDF1 BMP7	(i)SDF1 = 100 ng/mL (ii) BMP7 = 100 ng/mL	**In-vivo** Implantation of incisor scaffold orthotopically following mandibular incisor extraction in rat, and a human molar scaffold ectopically into the dorsum. 1.H&E staining 2. VK staining. 3. Quantification of average areal cell density and blood vessel numbers in coronal, middle, and apical thirds of the rat incisor scaffolds.	Whole Tooth regeneration	Anatomically shaped (a) Rat Mandibular Central Incisor (b) Human Permanent Mandibular First Molar	Specified dimensions of Rat Mandibular Central Incisor and Human Permanent Mandibular First Molar	(a) (b)

**Abbreviations**: Polyurethane (PU); Polyhedral oligomeric silsesquioxane (POSS); Bone mineral density (BMD); Bone volume fraction (BVF); Hydroxyapatite/Polylactic Acid (HA/PLA); Dental pulp stem cells of Dog (dDPSCs); Stromal-derived factor-1 (SDF1); Bone morphogenetic protein-7 (BMP7); Hematoxylin and eosin (H&E) staining; von Kossa (VK) staining.

## 7 Conclusion

Currently, numerous practitioners are turning to tissue engineering strategies to restore damaged or compromised tissues. These methods strive to rebuild tissues that are not only fully functional but also fully developed. The process of bioprinting, which fuses engineering principles with the fundamental principles of biology, is employed to fabricate three dimensional patterns that encompass all components of tissues, including cells and matrices. This technique has demonstrated remarkable success in generating both functional and intricate tissues, particularly those found within the oral and craniofacial region. These achievements encompass the creation of structures such as the periodontium, vascular and neural networks, the TMJ, and whole teeth. The feasibility of these feats owes itself to the construction of multiphasic scaffolds through the application of bioprinting. By enabling tissue healing to take place in distinct segments, these multiphasic scaffolds ultimately lead to the development of a unified structure. This capability empowers various practitioners to orchestrate and replicate crucial spatiotemporal occurrences throughout the wound healing process, paving the way for a foreseeable rejuvenation of multiple intricate tissues. Nevertheless, a significant portion of these discoveries remains confined to laboratory experimentation stages, necessitating validation through extensive animal experimentation and human clinical trials. Furthermore, researchers should give precedence to the integration of various bioprinting concepts with tissue engineering methodologies, including cell homing, stem cells, gene therapy, and other innovations. This synergy is essential to fully unlock the boundless potential of this cutting-edge technology.

## Declaration of Competing Interest

Declaration of Competing Interest: The authors declare that they have no known competing financial interests or personal relationships that could have appeared to influence the work reported in this paper.
